# 5A Syndrome

**Published:** 2020-01-05

**Authors:** Divya Nagabushana, Praveen-Kumar Srikanteswara

**Affiliations:** Department of Neurology, Bangalore Medical College and Research Institute, Bengaluru, India

**Keywords:** Allgrove Syndrome, Muscular Atrophy, Polyneuropathies

A 13-year-old boy presented with a long history of swallowing difficulty, regurgitation, and reflux with vomiting. He had undergone multiple surgeries for reflux and achalasia. He had history of alacrimia from early childhood. There was a history of recurrent syncopal episodes. He had severe wasting, and was undernourished with diffuse hyperpigmentation. On examination, he had postural hypotension, microcephaly, long philtrum with thin upper lip and atrophic tongue (Figure 1). Neurological examination revealed optic atrophy, absent gag, brisk jaw jerk, spastic tongue, and limb muscular amyotrophy with pyramidal signs. His serum cortisol level was low and serum thyroid stimulating hormone was normal. Brain magnetic resonance imaging (MRI) revealed pituitary hypoplasia. Nerve conduction study (NCS) revealed bilateral sensorimotor axonal neuropathy. He was started on cortisol replacement therapy.

The presence of amyotrophy (bulbospinal) and autonomic neuropathy with alacrimia, achalasia, and adrenal insufficiency clinched the diagnosis in this case, Allgrove syndrome or 5A Syndrome. Allgrove syndrome, typically known as triple A syndrome, consists of a triad of achalasia cardia, alacrima, and adrenal hypoplasia. It was first reported by Allgrove et al. in 1978.^1^ Patients with Allgrove syndrome can present with varied neurological manifestations such as autonomic neuropathy, sensorimotor axonal neuropathy, amyotrophy, optic atrophy, intellectual disability, seizures, and cranial nerve involvement.^2^ Early diagnosis and timely replacement of steroids can allow normal growth and development in pediatric patients.

**Figure 1:**
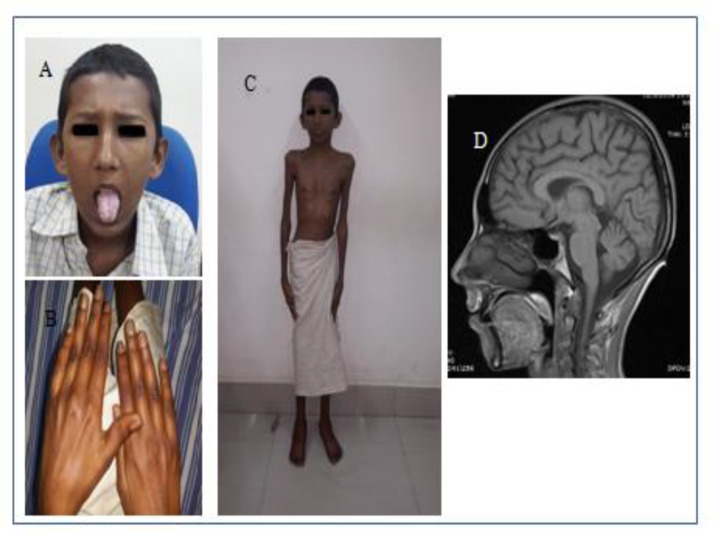
Image of the boy displaying the spastic atrophic tongue (A), hyperpigmented skin and thin wasted limbs and torso (B, C) and brain magnetic resonance imaging (MRI) T1W sagittal view showing pituitary hypoplasia (D)

## Notes:

**How to cite this article:** Nagabushana D, Srikanteswara PK. 5A Syndrome. Curr J Neurol 2020; 19(1): 43-4.
